# Preliminary Evaluation of the Application of Algae-Based Biostimulants on Almond

**DOI:** 10.3390/plants11223083

**Published:** 2022-11-14

**Authors:** Ivo Oliveira, Sílvia Afonso, Luís Pinto, Sofia Vieira, Alice Vilela, Ana Paula Silva

**Affiliations:** 1 Centre for the Research and Technology of Agro-Environmental and Biological Sciences (CITAB), Institute for Innovation, Capacity Building and Sustainability of Agri-Food Production, Inov4Agro, University of Trás-os-Montes e Alto Douro, Quinta de Prados, 5000-801 Vila Real, Portugal; 2 University of Trás-os-Montes e Alto Douro, Quinta de Prados, 5000-801 Vila Real, Portugal; 3 Chemistry Research Centre (CQ-VR), Biology and Environment Department, School of Life Sciences and Environment, University of Trás-os-Montes e Alto Douro, Quinta de Prados, 5000-801 Vila Real, Portugal; 4 Department of Agronomy, Centre for the Research and Technology of Agro-Environmental and Biological Sciences (CITAB), Institute for Innovation, Capacity Building and Sustainability of Agri-Food Production, Inov4Agro, Universidade de Trás-os-Montes e Alto Douro, 5000-801 Vila Real, Portugal

**Keywords:** *Prunus dulcis*, biostimulant, bioactive compounds, antioxidant activity, chemical composition, sensorial analysis

## Abstract

To improve almond performance under water limitations, the use of algae-based biostimulants may become a useful tool to reduce drought stress. However, besides possible effects on plant behavior, changes in fruit characteristics must also be considered. In this work, a preliminary study on the effect of two levels of an *Ascophyllum nodosum*-based biostimulant in the chemical characteristics of fruits from rain-fed cv. Marinada almond trees was carried out. The use of the recommended manufacturer’s dosage resulted in a decreased content of soluble sugars and proteins when compared to the use of half the recommended dosage and the control assays (water only). Similarly, and although no significant differences were recorded, the content of bioactive compounds (ortho-diphenols, total phenolics, and flavonoids) tended to increase in non-treated trees. Finally, sensory analysis of treated and non-treated fruits did not record any changes in the perceived attributes, showing that no negative effects on consumers’ acceptance will be caused by the application of this product. Long-term studies are needed to further confirm these results, also aimed at the monitoring of leaf gas exchange and water status parameters of trees.

## 1. Introduction

Nuts, including almonds, are receiving a new interest, linked to their chemical composition, which is known to provide some health benefits [1]. This has led to an increase in their production, creating also higher yields per area. However, the global pressure to reduce chemical inputs is forcing research into novel strategies and/or products, in which biostimulants are becoming ever more important [2]. They exercise their function by regulating several plant mechanisms, including carbon (C) and nitrogen (N) metabolism, enhancing antioxidant defenses and secondary metabolite production, increasing photosynthetic activity or improving water relations, among others [3]. In addition to their effects, due to their bio-based origin, impacts on biodiversity, environment, human health, and economy are lower than those found for inorganic and organo-mineral fertilizers [4]. The use of algae-based biostimulants is known to exert positive effects, either looking at crop yield or quality, but also for plant growth and performance, tolerance to environmental stress, nutrient uptake from soil or increase in antioxidant activity. However, and as for many other crops, the chemical composition of almonds can vary depending on several factors. Here can be included edaphoclimatic conditions, cultivar, or agricultural inputs, namely, fertilization [5], including biostimulants. Hence, this study aimed to provide a preliminary evaluation of how fertilization, using an algae-based biostimulant, affects the chemical and sensorial parameters of almonds.

## 2. Results and Discussion

This work intends to be a preliminary study of the effects of algae-based biostimulants in an important crop—almonds, although we are aware that other factors might influence the results (edaphoclimatic conditions, orchard location, and management), and that must be properly addressed in forthcoming studies. 

### 2.1. Bioactive Composition

**Figure 1:**
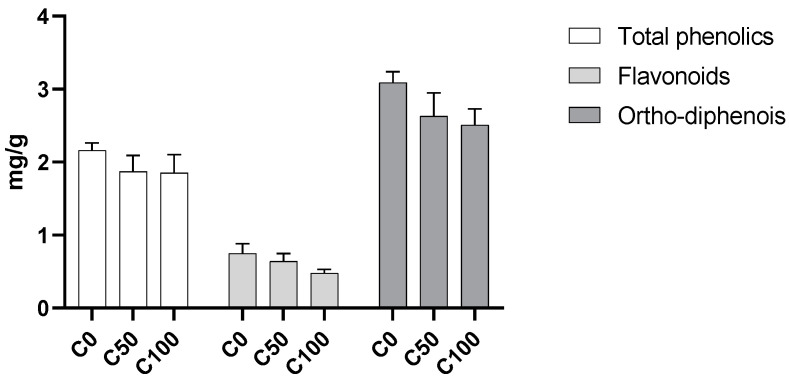
Total phenolic content (mg GAE/g), total flavonoid content (mg CE/g) and ortho-diphenols content (mg CAE/g DW). C0—control treatment with water; C50, Superfifty at 0.5 L/ha; C100, Superfifty at 1 L/ha.

No significant effect was recorded on the content of total phenolics, flavonoids, or ortho-diphenols (Figure 1). Even so, a clear tendency is visible, with a decrease in all values as the applied dosage of biostimulant increases. 

The values are within the range found for almonds [6], even though data regarding cv. Marinada, which is scarce to our knowledge, indicates a much higher content of total phenolics (19 mg gallic acid equivalents—GAE/g DW) [7] than the one recorded in the present work. The observed increasing trend with the reduction in dosage might be linked to an increase in stress in those trees. Indeed, other works have pointed out that almonds from trees under water or temperature stress present a higher amount of phenolic compounds [8,9], pointing out that biostimulants reduce the negative impact of water shortages or high temperatures. Flavonoids and ortho-diphenols follow the same trend registered for total phenolics, as well as a decrease with the application of biostimulants. Flavonoid content is within the range recorded for other almond cultivars grown in Portugal [10] and for what is usual for almonds [11,12]. For cv. Marinada, the available values for flavonoids indicate around 1.1 mg catechin equivalents—CE/g—and, for ortho-diphenols, around 4.7 mg caffeic acid equivalents—CAE/g [7]—with both being similar to the ones recorded in the present work. Flavonoids and ortho-diphenols, such as other phenolic compounds, are involved in the defense mechanisms of plants against stress, acting as antioxidants to offset oxidative stress [13]. Therefore, the increase in such compounds in trees without the use of biostimulants may be related to the defense-related functions of phenolic compounds [14].

### 2.2. Antioxidant Activity

The antioxidant activity of almonds with or without biostimulant application was evaluated using four different methodologies: ABTS, DPPH, FRAP, and the *β*-carotene bleaching assay. The analysis of the results shows that, in general, almonds have superior antioxidant activity in the samples without the use of biostimulants. 

**Figure 2:**
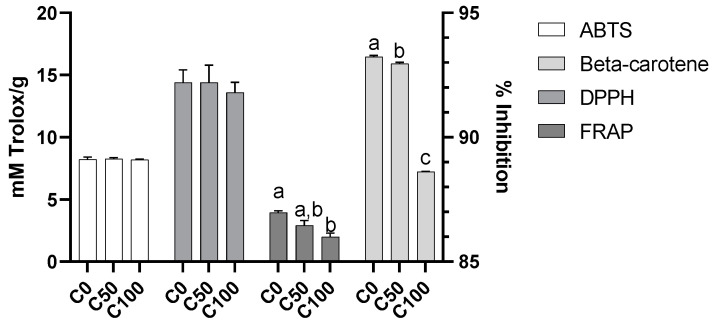
Antioxidant activity was recorded using the ABTS, DPPH, and FRAP assays (all mM Trolox/g) and *β*-carotene bleaching assay (% inhibition). Different small letters (a–c) indicate significant differences at *p* < 0.05 according to the analysis of variance (ANOVA) and multiple range test (Tukey’s test). C0—control treatment with water; C50, Superfifty at 0.5 L/ha; C100, Superfifty at 1 L/ha.

Significant differences between treatments were only recorded for the FRAP and *β*-carotene assays (Figure 2), and, in both methods, the higher results were found for the C0 samples. Independently of the method used, the recorded values (between 88.6% to 93.2%, for *β-*carotene, and between 2.01 and 3.95 mM Trolox/g for FRAP assay) can be considered similar to previous values found for almonds grown in Portugal [10,15]. For the ABTS and DPPH assays, similar values have been recorded by our groups in other almond cultivars [10,15]. Correlations were only found between the ortho-diphenol content and the antioxidant activity recorded in the FRAP methodology (R^2^ = 0.47, y = 1.422x − 0.938). Correlations between bioactive composition and antioxidant activity have been found elsewhere, as well as the absence of such correlations [10,15,16,17,18,19].

### 2.3. Chemical Composition

**Figure 3:**
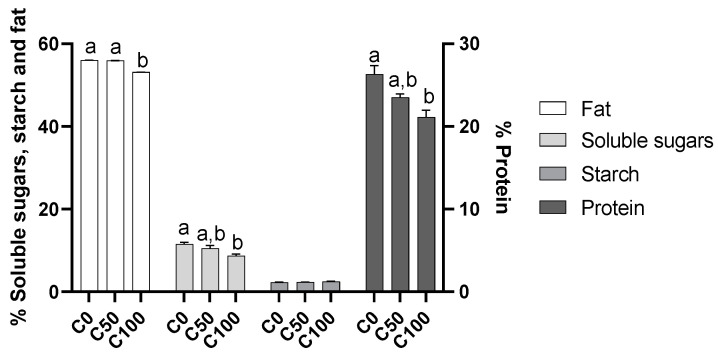
Chemical composition of almonds with or without the application of biostimulants. Different small letters (a,b) indicate significant differences at *p* < 0.05 according to the analysis of variance (ANOVA) and multiple range test (Tukey’s test). C0—control treatment with water; C50, Superfifty at 0.5 L/ha; C100, Superfifty at 1 L/ha.

Fat content ranged from 53.1% in the C100 almond to 56.0% in C0 samples (Figure 3), with significant differences recorded between C100 and the remaining two almond samples. These values are usual for almonds [5]. The significant decrease recorded in C100 samples might be linked to a reduction in water stress. Indeed, the work of Sakar et al. [20] showed that increased water stress resulted in an increased content of oil in almonds.

The content of soluble sugars, between 4.4% (C100) and 5.8% (C0), is within the range for almonds [21], as well as for this specific cultivar [22]. The soluble sugar of almonds decreased with the use of the biostimulant (Figure 3), being significantly different from C0 and C100. These results follow a different trend to that recorded previously by Pascoalino et al. [23], who found an increase in sugars with the application of biostimulants. Other authors suggest an increase in the sugar content with the imposition of water stress [23], which would indicate that the reduction that occurs with the application of biostimulants in the present work is linked to stress reduction in the almond trees. The accumulation of sugars works as a mechanism of the osmotic adjustment of plants under water-stress conditions [8,24].

Starch content, ranging between 1.13% (C0) and 1.24% (C100), is equivalent to what is known for this cultivar [22] and the average content of almonds [21]. Even though, without recording significant differences, starch content follows the inverse trend to the one recorded for soluble sugars: an increase with the application of biostimulant. This might be linked to the fact that starch content can be considered a marker of water stress, since plants convert starch into soluble sugars for their osmotic adjustment [25] or to act as signaling molecules, to activate downstream components in the stress-response cascade [26].

Protein content varied between 21.1% (C100) to 26.3% (C0), similar to the usual content in almonds [21] and for cv. Marinada [21]. Previous results with the use of algae-based biostimulants in almond trees also showed a reduction in the protein content [23], a result recorded in the present work. However, increases in protein content have been linked to water stress [9], which might signal that the use of biostimulants reduced the deleterious effect of environmental stress. 

**Figure 4:**
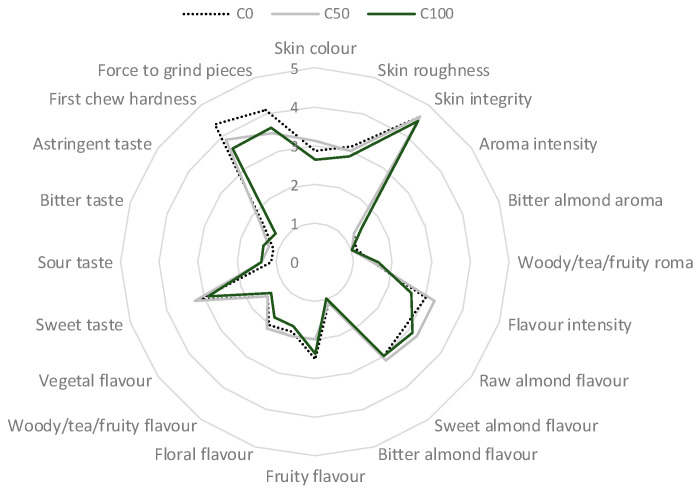
Spider plot of the sensory profile almond kernels with or without the application of biostimulants. C0—control treatment with water; C50, Superfifty at 0.5 L/ha; C100, Superfifty at 1 L/ha.

Finally, the sensory analysis performed by a panel of 12 trained panelists showed a sensory profile similar in all samples (Figure 4), indicating that the application of biostimulant did not cause any deleterious effect on the perceived attributes.

## 3. Materials and Methods

### 3.1. Samples

Almond (*Prunus dulcis* (Mill.) D.A. Webb) samples were obtained from an experimental field located in Freixo de Numão, Northeast of Portugal. Almond samples belong to cv. Marinada, from 5-year-old trees, grown in a rainfed orchard. Three treatments were applied: (i) Control—C0 (performed with water); (ii) Superfifty at 0.5 L/ha—C50 (BioAtlantis), a seaweed extract of *Ascophyllum nodosum*); (iii) Superfifty at 1 L/ha—C100, manufacturer’s recommended dosage, each applied two times, one at the BBHC phenological stage 81 and the second application at the BBHC phenological stage 85. Foliar spraying was conducted using a backpack sprayer. Samples from each treatment were harvested at commercial maturity, subdivided into three replicates, and deshelled to obtain raw kernels which included the skin.

### 3.2. Preparation of Extracts for Bioactive Compounds and Antioxidant Assays

Extracts were prepared by weighing 40 mg of finely ground samples and thoroughly vortex-mixing with 1 mL of methanol 70%. These mixtures were heated at 70 °C for 30 min, centrifuged at 13,000 rpm, and 1 °C for 15 min (Eppendorf Centrifuge 5804 R, Eppendorf AG, Hamburg, Germany), with supernatants, collected and filtered with Spartan filters (0.2 mm) to HPLC amber vials.

### 3.3. Bioactive Compounds: Ortho-Diphenols, Total Phenolic and Total Flavonoid Content

The methodology of Singleton and Rossi [27] was used for the quantification of total phenolics, with minor modifications: 20 µL of extract were mixed with 100 µL of Folin Ciocalteu’s phenol reagent (1:10 in bidistilled H_2_O) and 80 µL of 7.5% Na_2_CO_3_ in a 96-well microplate (Multiskan™ FC Microplate Photometer, Waltham, MA, USA). After incubation for 15 min at 45 °C, in the dark, absorbance values against a blank were recorded at 765 nm in a microplate reader (Multiskan GO Microplate Spectrophotometer, Thermo Scientific, Vantaa, Finland). A standard curve with gallic acid at different concentrations was performed and total phenolics content results were expressed as mg gallic-acid equivalent (GAE)/g of dry weight (DW) as the mean ± standard deviation (SD) of three replicates.

The colorimetric method described in Dewanto et al. [28], with some modifications, was employed for total flavonoid content determination. In a 96-well microplate, 25 µL of the extract was mixed with 100 µL of ultra-pure water, and 10 µL of 5% NaNO_2_. After 5 min in the dark, 15 µL of 10% AlCl_3_ was added and again the microplate was incubated at room temperature in the dark for 6 min. Then, 50 µL of NaOH 1 M, and 50 µL of ultra-pure water were added. The absorbance values were measured against a blank at 510 nm, with a standard curve of catechin at different concentrations performed to quantify total flavonoid content (values were expressed as mg catechin equivalent (CE)/g DW as the mean ± standard deviation (SD) of three replicates).

The method used for the determination of ortho-diphenols was adapted from Garcia et al. [29]. Twenty µL of extract were mixed with 100 µL of ultra-pure water, 80 µL of phosphate buffer (pH 6.5, 0.1 M) and 160 µL of 5% sodium molybdate (Na_2_MoO_4_·2H_2_O) solution. After 15 min in the dark, the absorbance was measured at 370 nm against a blank reagent, with caffeic acid used as the standard to prepare a calibration curve, and ortho-diphenolic content expressed as caffeic acid equivalents per g of dry sample (mg CAE/g DW) as the mean ± standard deviation (SD) of three replicates.

### 3.4. Antioxidant Activities

The method of Re et al. [30] was used to evaluate the 2,2-azino-bis(3-ethylbenzothiazoline-6-sulfonic) acid (ABTS) radical scavenging activity. An ABTS radical solution was prepared by mixing 7 mM of ABTS at pH 7.4 (5 mM NaH_2_PO_4_, 5 mM Na_2_HPO_4_, and 154 mM NaCl) with 2.5 mM K_2_S_2_O_8_. After overnight incubation in the dark at room temperature, this ABTS solution was diluted with ethanol until an absorbance of 0.70 ± 0.02 units at 734 nm was obtained. In each microplate well, 15 µL of the extract was mixed with 285 µL of freshly prepared ABTS solution, incubated at room temperature in the dark for 10 min, and absorbance values were measured at 734 nm. ABTS activity was expressed using the linear calibration curve of Trolox as g Trolox equivalent/g of fresh weight (FW).

The 2,2-diphenyl-1-picrylhydrazyl (DPPH) antioxidant activity assay was performed as described by Siddhraju and Becker [31]. Briefly, 20 µL of extract and 280 µL of freshly prepared methanolic radical DPPH solution (6 × 10^−5^ mol/L) were mixed in a 96-well microplate. The microplates were left for 30 min at room temperature and in the dark. The reduction in absorbance was measured at 517 nm and the DPPH activity was expressed using the linear calibration curve of Trolox as g Trolox equivalent/g f.w.

The ferric reducing activity of plasma (FRAP) assay was performed as described by Stratil, Klejdus, and Kubáň [32]. Briefly, a volume of 10 mM solution of 2,4,6-Tri(2-pyridyl)-S-triazine (TPTZ) in 40 mM HCl was mixed with the same volume of 20 mM FeCl_3_·6H_2_O and 10 times the volume of acetate buffer pH 3.6 (3.1 g sodium acetate and 16 mL acetic acid per L). 275 µL of the Fe^3+^-TPTZ mixture was added with 250 µL of the extract and incubated. After 5 min, the absorbances were recorded at 593 nm, and FRAP was expressed from the linear calibration curve of Trolox as μg Trolox equivalent/g f.w.

The *β*-carotene-linoleic acid bleaching assay was performed using the method described by Salleh et al. [33]: a mixture of *β*-carotene and linoleic acid was prepared by adding 0.5 mg of β-carotene, 1 mL chloroform (HPLC grade), 25 µL linoleic acid and 200 mg Tween 40. After completing under-vacuum evaporation of the chloroform, 100 mL of water was added to the residue and gently stirred to form a yellowish emulsion. 50 µL of extract were mixed with 0.25 µL of the yellowish emulsion and incubated in a water bath at 50 °C for 2 h followed by the measurement of absorbance values at 470 nm against a blank. Percentage inhibition (I%) of lipid peroxidation was calculated using the following Equation:

I% = (A after incubation/A before incubation) × 100(1)

where A after incubation is the absorbance value of *β*-carotene after 2 h of incubation and A before incubation is the absorbance value of *β*-carotene before incubation.

## 4. Conclusions

This preliminary evaluation points out some effects of the use of algae-based biostimulants in almonds, specifically the reduction in the content of bioactive compounds and antioxidant activity, but also of sugars and protein. Even so, the continuation of this study, as well as others, focusing on plant parameters, such as leaf gas exchange or water status, must be conducted, providing further insight into biostimulants’ effects on almond crops.

## Data Availability

The data presented in this study are available on request from the corresponding author.
